# Transvaginal ultrasound diagnosis of colouterine fistula secondary to sigmoid colon cancer: A case report

**DOI:** 10.1016/j.radcr.2026.06.029

**Published:** 2026-07-03

**Authors:** Yan Yi, Ling Wang, Dan Fei, Tao Wang, Shan Luo, Zhi He Wang, Gang Bai

**Affiliations:** a Department of Ultrasound Medicine, The Eighth Hospital of Wuhan, Wuhan, China; b Department of Gastroenterology, Yueyang Central Hospital, Yueyang, China; c Department of Dermatology, The First People’s Hospital of Tianmen, Tianmen, China; d Department of Ultrasound Medicine, The First Affiliated Hospital of Yangtze University, Jingzhou, China

**Keywords:** Colouterine fistula, Transvaginal ultrasound, Sigmoid colon cancer, Pelvic fistula, Dynamic imaging

## Abstract

Colouterine fistula is an exceptionally rare pelvic fistula, typically arising from diverticulitis, malignancy, or trauma.‌ The thick uterine myometrium serves as a natural barrier, rendering direct intestinal invasion of the uterine wall unlikely and thus complicating preoperative diagnosis. ‌Dynamic real-time imaging plays a pivotal role in evaluating such lesions. This case report describes a 74-year-old woman patient who presented with a 1-month history of vaginal flatus and fecaloid discharge. The symptoms were caused by a colouterine fistula secondary to a sigmoid colon malignancy. Gynecological examination confirmed the presence of vaginal flatus and fecal-like discharge. Transvaginal ultrasound revealed a 3 mm wide discontinuity in the left myometrial wall of the uterus, communicating with the sigmoid colon. Dynamic observation demonstrated intestinal gas entering the uterine cavity through the fistula, accompanied by a mobile air-fluid interface and color Doppler twinkling artifact. CT and MRI further confirmed sigmoid colon tumor invasion of the uterine wall with fistula formation. ‌Intraoperatively, the diagnosis was confirmed as sigmoid colon cancer secondary to colouterine fistula.‌ Owing to extensive tumor invasion, only a double-barrel transverse colostomy was performed. The patient experienced an uncomplicated postoperative recovery. ‌This case underscores the value of transvaginal ultrasound in early diagnosis, as it enables noninvasive, dynamic visualization of fistula morphology and gas-liquid flow, serving as a critical adjunct to CT/MRI.

## Introduction

Colouterine fistula is a rare pelvic internal fistula, most commonly secondary to diverticulitis, colorectal malignancy, and pelvic surgery complications [[1], [2], [3]]. Due to its concealed clinical presentation, diagnosis is often delayed, and some cases are not diagnosed until intraoperatively [1]. CT and MRI are the primary imaging methods, capable of demonstrating intrauterine air, bowel wall thickening, and the fistula tract, but are limited to static imaging [4,5]. Transvaginal ultrasound offers real-time, dynamic, and radiation-free advantages, showing potential in assessing fistula activity. This case report presents a colouterine fistula secondary to sigmoid colon cancer, highlighting the dynamic features of transvaginal ultrasound (e.g., air passage through the fistula into the uterine cavity) in comparison with CT, MRI, and surgical findings, to demonstrate its value in early diagnosis.

## Case presentation

The patient is a 74-year-old woman who presented with a 1-month history of vaginal flatus and fecal-like discharge. She reported no prior abdominal surgery, pelvic inflammatory disease, or malignancy. Physical examination demonstrated mild lower abdominal tenderness but no palpable masses. The patient denied recent trauma and reported no systemic symptoms, including fever, worsening abdominal pain, or unintentional weight loss. Blood tests revealed elevated inflammatory markers, and vaginal microbiota analysis showed alterations in the microbial flora and acidic environment.

Transvaginal ultrasound (Mindray endocavitary probe) demonstrated an anteverted uterus with well-defined margins and normal dimensions. Multiple gas-like hyperechoic foci were visualized within the uterine cavity, extending from the fundus to the internal cervical os (Video Editing S1 and S2). The myometrium in the left uterine wall exhibited a focal discontinuity, forming a 3 mm wide tract communicating with the adjacent sigmoid colon; the margins of this communication were ill-defined. Dynamic observation revealed passage of intestinal gas through the tract into the uterine cavity, creating a mobile gas-fluid interface (Fig. 1 and Video Editing S3). Color Doppler imaging showed a flickering signal within the fistula tract and rich intestinal blood flow, suggesting local inflammatory changes. Ultrasound impression: Abnormal uterine–intestinal communication, suspicious for colouterine fistula.

**Fig. 1:**
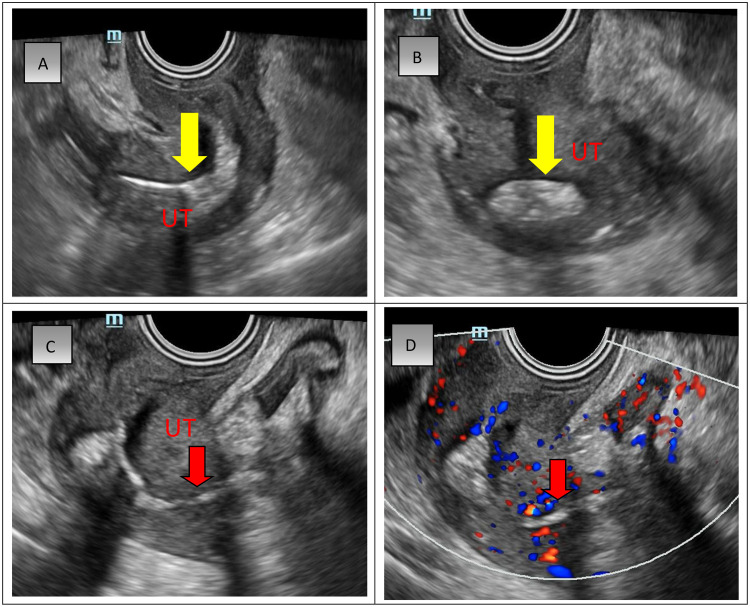
(A) Longitudinal transvaginal ultrasound view of the uterus; (B and C) transverse transvaginal ultrasound views of the uterus; (D) transverse transvaginal ultrasound view of the uterus with color Doppler imaging. The yellow arrow denotes gas within the uterine cavity, the red arrow indicates the sinus tract, and UT stands for uterus.

CT and MRI findings‌: CT demonstrates thickening of the sigmoid colon wall and an irregular soft tissue mass at the mid-to-lower segment, with an involved length of approximately 7 cm, infiltrating the anterior wall of the uterus. Gas is observed within the uterine cavity (Fig. 2). MRI further confirms that the sigmoid colon tumor has penetrated the left anterior superior myometrium and left lateral myometrium, forming a fistula with indistinct boundaries, indicating local invasion (Fig. 3). Imaging staging: T4bNxMx and EMVI (+).

**Fig. 2:**
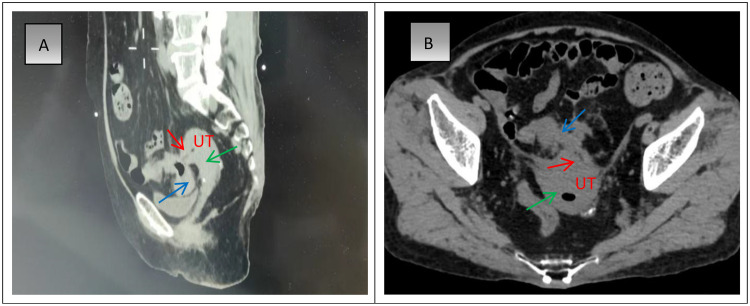
(A) Sagittal noncontrast computed tomography (CT) view; (B) axial noncontrast computed tomography (CT) view. The red arrow indicates the sinus tract, the green arrow denotes the uterus, the blue arrow points to the sigmoid colon tumor, and UT stands for uterus.

**Fig. 3:**
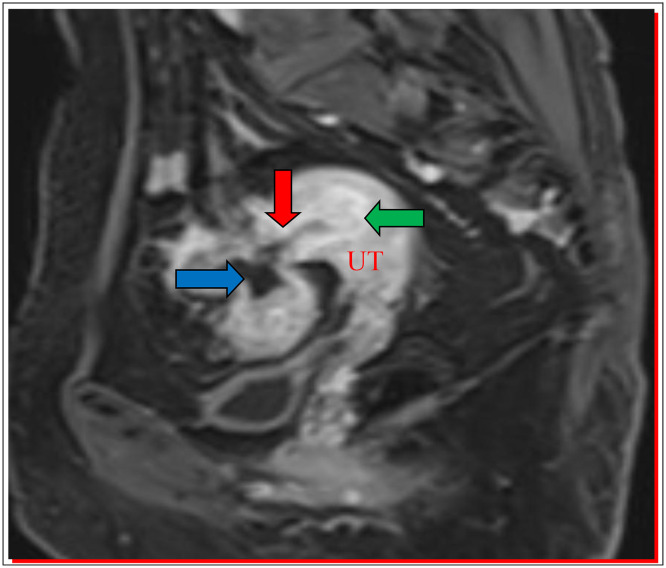
Sagittal magnetic resonance imaging (MRI) view. The red arrow indicates the sinus tract, the green arrow denotes the uterus, the blue arrow points to the sigmoid colon tumor, and UT stands for uterus.

Operative and follow-up summary: Intraoperatively, a tumor measuring approximately 5 cm in diameter was identified in the distal sigmoid colon. It was densely adherent to the anterior uterine wall, with local tissue destruction forming a fistula. Radical resection was not performed due to tumor invasion of the left ureter and iliac vessels. Instead, a double-barrel transverse colostomy was conducted. Pathologic examination confirmed adenocarcinoma (Stage T4N1M0). Postoperatively, the patient developed incisional fat liquefaction and infection, which were successfully managed with antibiotics and local wound care. The patient recovered well with normal stoma function. Following discharge, she received adjuvant chemotherapy. At the 6-month follow-up, there were no signs of recurrence.

## Discussion

Colouterine fistula is an extremely rare complication of colorectal disease. Its low incidence is closely associated with the thick, robust structure of the myometrium, which serves as a natural anatomical barrier. This barrier makes direct invasion of the uterus by intestinal pathology difficult, resulting in a significantly lower occurrence compared to colovesical or colovaginal fistulas [6]. Etiologically, the condition is primarily classified into 4 categories: inflammatory (e.g., diverticulitis, Crohn’s disease, and ulcerative colitis) [1], iatrogenic (colorectal surgery and intrauterine procedures, etc.) [2], neoplastic (e.g., colorectal cancer and endometrial cancer) [3], and radiation-induced injury [7]. Among these, diverticulitis is the most common cause, particularly in elderly patients. It is noteworthy that while malignant tumors—especially sigmoid colon cancer—can cause colouterine fistulas, their actual occurrence is even rarer due to the need to breach the aforementioned anatomical barrier. When they do occur, it often indicates locally advanced disease, carrying significant clinical implications [3]. The present case represents such a rare scenario, in which sigmoid colon cancer directly infiltrated the uterine myometrium and formed a fistula during its progression. Despite being the most common gastrointestinal malignancy—a disease whose development is influenced by multiple factors including genetics, environment, and chronic inflammation [8] and which can directly invade adjacent organs at locally advanced stages [9]—colorectal cancer rarely results in colouterine fistula in clinical practice, owing to the effective protective barrier of the uterine myometrium.

Due to its rarity, the clinical diagnosis of this condition poses significant challenges. On the 1 hand, the clinical presentation of this condition is closely linked to its underlying etiology and pathology. Typical symptoms include vaginal flatus or the discharge of fecal or purulent material, which may persist for days to months. In contrast, systemic symptoms such as fever and abdominal pain are often minimal or entirely absent. This pattern frequently results in missed diagnosis or misdiagnosis as other common gynecological or gastrointestinal disorders. On the other hand, because fistulous tracts are often minute, conventional imaging modalities have limited detection capability, frequently resulting in diagnostic difficulties and delays prior to surgery. Therefore, in clinical practice, maintaining a high index of suspicion in patients with relevant symptoms—particularly unexplained fecal vaginal discharge—is crucial.

Currently, various imaging modalities have been employed for diagnosing such fistulas. However, this is the first reported case in which a fistula between the sigmoid colon and the uterus was visualized via transvaginal ultrasound. The characteristic transvaginal ultrasound findings in this case comprised: (1) focal discontinuity of the uterine wall, (2) hyperechoic gas within the uterine cavity, (3) ill-defined echogenic borders between the uterus and adjacent bowel accompanied by disorganized bowel echotexture and increased vascular flow as indirect signs, and (4) the direct sign of gas movement within the fistula tract with a flickering signal on color Doppler. Computed tomography (CT) can demonstrate gas within the uterine cavity communicating with the colonic wall [4]. Magnetic resonance imaging (MRI), particularly on T2-weighted sequences, clearly depicts a low-signal fistula tract, showing colouterine adhesion and communication, thereby suggesting the diagnosis [5]. Hysterosonography also aids in delineating the fistula and adjacent anatomy [10]. In contrast, modalities such as colonoscopy or barium enema have limited sensitivity for detecting small colouterine fistulas [11]. While the charcoal provocation test may indicate communication, it cannot localize the tract or define its anatomy [11]. Although several imaging techniques can reveal a fistulous connection, the definitive confirmation in this case came from the real-time transvaginal ultrasound visualization of intestinal gas entering the uterine cavity via the fistula. This finding underscores the unique diagnostic value of transvaginal ultrasound for rare pelvic fistulas. To date, reports describing the transvaginal ultrasound diagnosis of malignancy-associated colouterine fistulas are exceedingly rare. This preoperatively confirmed case underscores the significant clinical utility of transvaginal ultrasound in the early identification and evaluation of such fistulous tracts.

Although most cases of colouterine fistula require surgical intervention, with various techniques having been reported, the overall management strategy must be individualized based on the underlying etiology. Owing to the rarity of malignancy-associated colouterine fistulas, no standardized treatment protocol has been established. For such cases, en bloc tumor resection with fistula repair is the standard approach, aimed at achieving complete oncological clearance and restoring anatomical integrity [4]. Adjuvant chemotherapy should be administered as clinically indicated. Halevy et al. [12] reported favorable outcomes following en bloc resection in 2 patients with malignant colouterine fistulas. In contrast, due to extensive tumor invasion, the patient in the present case was not a candidate for radical surgery. Instead, palliative double-barrel transverse colostomy was performed, followed by chemotherapy for disease control. The management of benign fistulas is guided by their underlying etiology. For diverticulitis-associated fistulas, a 2-stage surgical approach is recommended. This involves initial resection of the affected bowel segment with diverting colostomy, followed by delayed anastomosis after resolution of inflammation, to minimize infection risk and optimize healing rates [13]. Fistulas due to other benign causes, such as iatrogenic injury, may be managed with simple repair, provided there is no active inflammation in the surrounding tissues. The core principle guiding treatment selection is thorough evaluation of the patient’s overall condition, tumor stage, and fistula anatomy, with management individualized after balancing surgical benefits and risks. Prognosis is dictated by the underlying etiology. Patients with benign fistulas have a low recurrence rate and overall survival that closely approximates that of the general population. Conversely, the prognosis for malignancy-associated fistulas is substantially poorer, being driven primarily by tumor stage, the presence of metastases, and response to therapy. In the present case, no recurrence was observed at the 6-month follow-up; however, long-term surveillance for tumor progression and fistula healing status remains necessary.

## Conclusion

In summary, a colouterine fistula—particularly one secondary to malignancy—is a rare and diagnostically challenging complication with an insidious clinical presentation. The combination of nonspecific symptoms (e.g., vaginal flatus, feculent, or foul‑smelling discharge) and the limited diagnostic yield of conventional imaging commonly contributes to delays in both diagnosis and clinical management. This case illustrates that transvaginal ultrasound, as a noninvasive, real‑time, and dynamic imaging modality, can directly visualize the passage of intestinal gas into the uterine cavity—a key diagnostic finding. It is particularly valuable for early detection and assessment in suspected patients, especially elderly women with a history of intestinal pathology, and may serve as a first‑line screening tool. Its findings not only offer crucial evidence for guiding further imaging (e.g., CT/MRI) but also help establish a framework for multidisciplinary collaborative management. Therefore, in the management of such conditions, clinicians should maintain a high index of suspicion, actively obtain targeted imaging for atypical symptoms, and advocate for a structured multidisciplinary team (MDT) approach involving colorectal surgery, gynecology, and radiology. Through resource integration, early diagnosis, individualized treatment, and standardized follow-up, clinical outcomes can be optimized and patient quality of life enhanced. This case provides a practical new perspective on managing this rare complication.

## Patient consent

Written informed consent was obtained from the patient for publication of this case report and any accompanying images.

## Appendix Supplementary materials

S1: Dynamic image 1 of the uterus by transvaginal ultrasonography.mp4. Image, video 1 dummy alt text

S2: Dynamic image 2 of the uterus by transvaginal ultrasonography.mp4. Image, video 2 dummy alt text

Dynamic image of sinus tract gas movement in the transverse section of the uterus by transvaginal ultrasonography.mp4 Video clip S1 and S2 are dynamic images of longitudinal and transverse sections of the uterus by transvaginal ultrasound; Video clip S3 is a dynamic image of gas movement in the sinus tract of the transverse section of the uterus by transvaginal ultrasound. Image, video 3 dummy alt text
